# TULP: A Trusted Upload and Linkage Procedure for Large-Scale Cohort Data and Statistics Netherlands Data

**DOI:** 10.23889/ijpds.v11i4.3813

**Published:** 2026-07-06

**Authors:** Camiel van der Laan

**Affiliations:** 1 Utrecht University, Utrecht, Netherlands

The microdata of Statistics Netherlands (CBS) can be accessed for research under strict privacy and security conditions and may be linked to external datasets within a secure remote-access environment. Until recently, these analyses were constrained by the limited computational capacity of the CBS infrastructure. The introduction of the ODISSEI Secure Supercomputer (OSSC) removes this barrier by enabling high-performance computing while maintaining the stringent confidentiality requirements of CBS data. A central component of this infrastructure is the Trusted Upload and Linkage Procedure (TULP), which facilitates the secure linkage of large-scale external data—such as genomic datasets—to CBS population register data. In TULP, SURF acts as a trusted third party, applying a pseudorandom record-shuffling protocol that prevents researchers from reconstructing personal identities while preserving linkage accuracy. The feasibility and security of this approach were demonstrated by de Zeeuw et al. (2021) and have since been further refined to comply with current regulatory and technical standards. This development now enables large-scale and computationally intensive linkage projects, including genome-wide association studies (GWAS) on CBS microdata. In this presentation, we outline the TULP framework, discuss its implementation, and demonstrate its potential for expanding the scope of large-scale, computationally intensive research in the Netherlands with a genome-wide association study of healthcare costs.

